# Association Between Job Stress and Organizational Commitment in Three Types of Chinese University Teachers: Mediating Effects of Job Burnout and Job Satisfaction

**DOI:** 10.3389/fpsyg.2020.576768

**Published:** 2020-10-08

**Authors:** Peng Wang, Pengpeng Chu, Jun Wang, Runsheng Pan, Yu Sun, Meng Yan, Longzhen Jiao, Xiangping Zhan, Denghao Zhang

**Affiliations:** ^1^Department of Psychology, Shandong Normal University, Jinan, China; ^2^Department of Psychology, Renmin University of China, Beijing, China; ^3^The Laboratory of Department of Psychology, Renmin University of China, Beijing, China

**Keywords:** job stress, organizational commitment, job burnout, job satisfaction, Chinese university teacher, the type of university

## Abstract

Utilizing the Job Demands-Resources (JD-R) model as the theoretical framework, this study examines the relationship between job stress, job burnout, job satisfaction, and organizational commitment among 1,906 university teachers in China, and investigates teachers’ differences across groups. The result of SEM indicates that job burnout and job satisfaction could play mediating roles between job stress and organizational commitment. The result of multi-group analysis shows that for national university teachers, the positive effect of job stress on job burnout is the highest among three types of university teachers, the negative effect of job burnout on organizational commitment is lower compared with provincial university teachers and the negative effect of job burnout on job satisfaction is lower compared with provincial university teachers. Only for provincial university teachers, the job stress can significantly positively predict organizational commitment, and the independent mediating effect of job burnout is significantly greater than job satisfaction. The practical advice to enhance Chinese university teachers’ organizational commitment was provided in the end.

## Introduction

Since teachers experience a great deal of stress during the course of their careers, teaching has long been considered a high-stress occupation (Johnson et al., 2005; Noor and Zainuddin, 2011; Liu and Cheung, 2015). For academic personnel, stressors include high pressure to publish and task-role balance management put extra burden on their shoulders (Graça et al., 2020). This situation can be even harsher for Chinese university teachers due to the ambition of building more world-class universities (Zhu et al., 2018; Zhang et al., 2019). According to a survey conducted among Chinese university teachers in 2013, more than 36% of young teachers reported that they were facing great stress, and job stress has been a critical negative factor affecting their job satisfaction (Liu and Zhou, 2016).

To understand the generation mechanism of job stress and its influence, we introduced the Job Demands-Resources (JD-R) model (Demerouti et al., 2001), which is an overarching model that may be applied in teacher group (Bakker et al., 2005) and Chinese culture context (Hu et al., 2016). The JD-R model posits that the characteristics of a job can be classified as either job demands or job resources, which are the antecedents of job burnout and engagement. In addition, this model assumes dual different underlying psychological processes of work: a health impairment process which may lead to burnout, while a motivational process that may lead to work engagement and organizational commitment (Bakker et al., 2003, 2005; Hu et al., 2016). Via burnout and work engagement, job demands and job resources have been shown to influence various other aspects of employees functioning (Van den Broeck et al., 2013), such as job satisfaction (Martinussen et al., 2007) and organizational commitment (Hakanen et al., 2008). Meanwhile, job resources could play a moderate role in the relationship between job stress and its psychological outcomes. For example, it can buffer the impact of job demands on burnout (Bakker et al., 2005). With the guidance of the JD-R model, we incorporated study variables well into a theoretical framework which includes the underlying mechanisms regarding how job stress influences organizational commitment, the mediating effect of job burnout and job satisfaction and the moderating effect of university types which represent major difference in organizational resources. Besides, our study also extended the JD-R model in the following two aspects. First, we revisited the relationship of job stress and organizational commitment in a sample of Chinese university teachers. Second, we introduced university types as a moderating effect which is scarcely investigated in the existing literature.

### Job Stress and Organizational Commitment

Job stress refers to any affect-laden negative experience that is caused by an imbalance between job demands and the response capability of the workers. When job demands are too high to cope with, stress reactions are likely to occur (Schaufeli and Enzmann, 1998). Teacher’s job stress was firstly defined as teachers’ negative experience due to their negative cognition of classroom environment (Kyriacou and Sutcliffe, 1977). Relevant research showed that people working in the helping professions, especially educators, are particularly prone to being stressed out (Rothmann and Barkhuizen, 2008), and have suffered higher levels of job stress than those working in many other institutions (Winefield and Jarrett, 2001). There is plenty of evidence suggests that academic staff from different countries have suffered above-average level of job stress to some extent (Abouserie, 1996; Yang and Chen, 2016).

Organizational commitment was defined as an employee’s identification and involvement in the organization (Porter et al., 1974). Committed individuals are characterized by sharing of values, desiring to maintain membership, and willing to exert effort on behalf of the organization. As for schools, committed teachers may have a strong psychological connection with their subject areas, students, and school (Firestone and Pennell, 1993). Numerous studies have indicated that the organizational commitment of school is closely related to teachers’ work attitude (Imran et al., 2014), work performance (Wang, 2010; Jing and Zhang, 2014), and turnover intention (Imran et al., 2017). Teachers’ organizational commitment has big impact on the efficiency and effectiveness of their work (Fresko et al., 1997; Louis, 1998) and is directly related to teacher’s job involvement and enthusiasm (Emami et al., 2013).

Job stress is thought to be one of the antecedents of organizational commitment, but understanding of the relationship between job stress and organizational commitment has been both inconsistent and incomplete (Abdelmoteleb, 2019). Although a large number of studies have reported a negative impact of job stress on organizational commitment (e.g., Jamal, 1990; Judeh, 2011; Myers, 2011), other studies have not supported this link (e.g., Parasuraman and Alutto, 1984; Elangovan, 2001; Antón, 2009). In the light of JD-R model, organizational commitment is used as an outcome that may be negatively influenced by burnout through the health impairment process (Hakanen et al., 2006; Llorens et al., 2006). Hu et al. (2011) found that the stress process via burnout led to negative organizational outcomes (low organization commitment, etc.) in two independent Chinese samples. Besides, high demands do not always lead to low organizational commitment. Bakker et al. (2010) found that employees endorse most positive work attitudes (task enjoyment and organizational commitment) when job demands and job resources are both high. Thus, whether job stress brings negative effect is not only related to job demands intensity, but also affected by other factors such as job resources.

In the past two decades, China has witnessed the reform of the employment system in Chinese higher education. Nowadays, new managerialism and academic capitalism have permeated in Chinese university institutions, which prompt the administration structure and performance evaluation system to be more academic productivity-oriented (Bao and Wang, 2012). Under such background, Chinese university teachers need to work harder to increase academic output, they may bear higher job demands in this process, it also means greater chance of promotion and higher salaries (namely high job resources) for hard-working employees, which may facilitate their organizational commitment. For instance, one research conducted in China revealed that perceiving higher job stress would present higher organizational commitment among university PE teachers (Kang and Liu, 2018). Thus, we assume there is positive predictive relation between job stress and organizational commitment in the sample of Chinese university teachers, which is different from the previous research conducted in western culture context.

Hypothesis 1: Job stress could positively predict organizational commitment.

### The Mediating Role of Job Burnout

University teachers are among the professionals most susceptible to burnout (Zhong et al., 2009). Job burnout is seen as a psychological syndrome that occurs in response to chronic work-related stressors (Maslach et al., 2001), which is a comprehensive manifestation of emotional exhaustion, depersonalization, and reduced personal accomplishment (Maslach et al., 1997; Wu et al., 2008; Leiter et al., 2014). Burnout occurs at work like depression occurs in private life, they share similar symptoms such as feeling of low energy and low self-esteem (Ahola et al., 2014). In the JD-R model, Job burnout could be the consequence of two health impairment processes: the exhaustion process caused by high job demands, and the process of failing to meet demands caused by lacking resources (Demerouti et al., 2001). The imbalance that teachers perceived between job demands and job resources affects their psychological well-being at work, which may develop into burnout (Prieto et al., 2008). Job burnout is viewed as a consequence of one’s exposure to chronic job stress (Maslach et al., 2001; Lloyd et al., 2009; Shirom et al., 2009): when short-term stress cannot be alleviated in time, job burnout occurs. Moreover, burnout in teachers is associated with absenteeism, turnover intention, low job satisfaction, negative attitudes and disinterest toward students and their education (D’Amico et al., 2020). Ha et al. (2011) found that emotional exhaustion and reduced personal accomplishment were negatively related to job satisfaction and organizational commitment in the group of South Korean PE teachers. In Chinese sample, studies also showed that university teachers with higher job stress would experience higher job burnout (Li, 2018), and job burnout was negatively related to organizational commitment (Zhou, 2015). Consistent with previous research, we propose that job burnout could play a mediating role between job stress and organizational commitment in Chinese university teachers’ group.

Hypothesis 2: Job stress could affect organizational commitment via job burnout.

### The Mediating Role of Job Satisfaction

Job satisfaction is an attitude or subjective experience toward an individual’s job (Miembazi and Qian, 2017). Study shows that there is a negative relationship between job stress and job satisfaction among university staff (Ahsan et al., 2009). For instance, study find that job stress is negatively related to job satisfaction among university teachers in Hong Kong (Leung et al., 2000). Meanwhile, job satisfaction and organizational commitment share a significantly strong positive relationship (Silva, 2006), and job satisfaction can be viewed as an antecedent of organizational commitment (Rusbult and Farrell, 1983; Nagar, 2012; Wang et al., 2016). In addition, job satisfaction could lead to increase in employees’ efficiency, commitment and decrease in absenteeism (Aziri, 2011). It is very important that employees are satisfied with their work so that they can be committed to their organizations (Aksoy et al., 2018). One study conducted under Arabic cultural context found that job satisfaction mediated the influences of role conflict and role ambiguity on various facets of organizational commitment (Yousef, 2013). For teaching occupation, teacher’s satisfaction with his or her job may have strong implications for his or her emotional attachment to the organization (Meyer et al., 2002). To be more specific, job stress influenced psychological health via the mediated effect of job satisfaction (Pan et al., 2010), and job satisfaction is a mediator between job stress and organizational commitment (Lu, 2007). In line with previous research, we indicate that job satisfaction could also play a mediating role between job stress and organizational commitment in Chinese university teachers’ sample.

Hypothesis 3: Job stress could affect organizational commitment via job satisfaction.

Previous scholars tended to view job burnout and job satisfaction as mediate variables since they represent cognition changes of different emotional polarity (Reizer, 2015). Studies have shown a significant negative relationship between job burnout and job satisfaction (Maslach and Schaufeli, 1993). Salehi and Gholtash (2011) noted a negative relationship between burnout and job satisfaction when studying the organizational citizenship behavior of academics. A study conducted by Høigaard et al. (2012) in Norway also observed a strong negative correlation between these two variables. In addition, Wolpin et al. (1991) found burnout appeared to be a cause of reduced job satisfaction in a longitudinal study with teachers and administrators. A study similar to our research conducted by Nagar (2012) which developed a linkage among burnout, job satisfaction, and organizational commitment among university teachers in Jammu’s sample found that burnout led to decreased job satisfaction, and high job satisfaction contributed increase in organizational commitment. Above all, the fourth hypothesis is brought up to explore the mechanism between job stress and organizational commitment among Chinese university teachers.

Hypothesis 4: Job stress could affect organizational commitment via the multiple mediated effect of job burnout and job satisfaction.

Additionally, since burnout is the first response to job stress, it reflects unremitting work demands, which is a result of accumulated work-related stress (Pines and Keinan, 2005). For example, previous research suggested that burnout would mediate the relationship among job stress, depressive symptoms, and low scores on physical health (Zhong et al., 2009). That is, compared with job satisfaction, job stress may be more closely related to job burnout. Thus, we hold that in the relationship between job stress and organizational commitment, the effect of job burnout is stronger than that of job satisfaction.

Hypothesis 5: The independent mediating effect of job burnout is greater than job satisfaction.

### The Moderating Role of University Types

According to the classification of administrative subordination, China has three types of public universities: national universities (refer to 211 or 985 project universities, resource-rich and top universities in China), provincial universities (resource-medium and inferior to national universities), and municipal universities (resource-poor and the lowest level among three types of universities). These three types of universities in China are similar to doctoral-granting institution, master-granting institution, and bachelor-granting institution in America separately (Li and Yi, 2010). In China, the resource differences among different university types are of great significance to teachers. Many Chinese scholars have regarded the university type as an indicator of possible difference in the study of university teachers, such as turnover intention (You, 2014) and academic output (Zhang and Xue, 2019). Notably, some research revealed the moderating effect of university types. One study conducted by Chinese scholars Wang and Zhang (2018) which investigated the negative moderating effect of university types on the relationship between university-industry collaboration and research performance in China. They divided universities into research oriented universities (211 or 985 project universities) and non-research oriented universities (non-211 or 985 project universities). Result indicated that organizational resources (i.e., research funds) difference is the key aspect to distinguish different university types. In the JD-R model, job resources may play a role in buffering the health-impairing impact of job demands (Bakker et al., 2007). In addition, job resources was commonly distinguished as both extrinsic and intrinsic aspect to the job (Bakker et al., 2003). For extrinsic, it may be located at the macro, organizational level (e.g., salary or wages, career opportunities, job security) (Demerouti and Bakker, 2011). Although few researchers have noticed the influence of university type, we hold that it reflects the organizational resources difference, which may bring moderating effect. Thus, we attempt to use multi-group analysis to test the differences among three types of university in our research model.

Hypothesis 6: University’s type could moderate the relationships among job stress, job burnout, job satisfaction, and organizational commitment.

**FIGURE 1:**
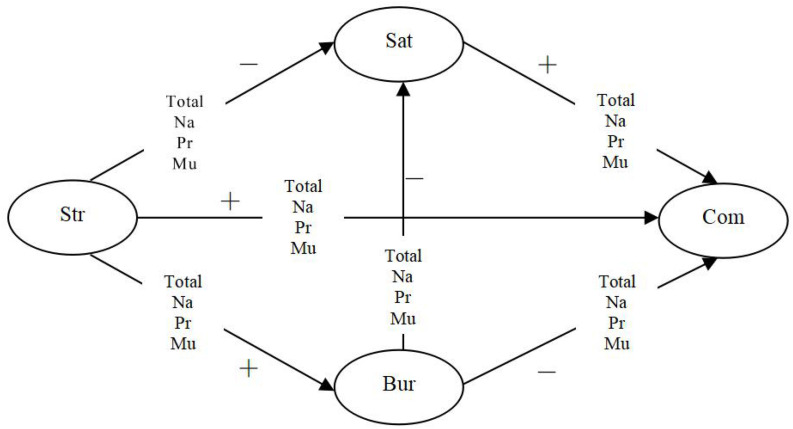
Conceptual model of the relationship among job stress, job burnout, job satisfaction, organizational commitment and the type of university. Na, national university; Pr, provincial university; Mu, municipal university; Str, job stress; Bur, job burnout; Sat, job satisfaction; Com, organizational commitment.

The proposed conceptual model is illustrated in Figure 1.

## Materials and Methods

### Participants and Procedure

Our data were obtained from a cluster sample, includes 2,200 teachers from 22 universities in China (encompassing 4 national universities, 10 provincial universities, and 8 municipal universities) by several questionnaires. The authors reached these participants through a contact teacher in each university, and all the contact teachers were trained with unified instruction before the formal test. After informing participants the anonymity of the research and obtaining participants’ agreement, measurement were conducted by trained contact teacher. Finally, all questionnaires were mailed to authors by the contact people.

As a result, 1,988 participants completed and returned the questionnaire, yielding a response rate of 90.4%; 82 observations were excluded because of incomplete information, leaving total of 1,906 observations (979 males, 924 females, 3 missing values) for empirical analysis. Most of the participants were highly educated (1,384 having a master’s or doctoral degrees) and were married (*n* = 1524); 233 were from national universities; 1155 came from provincial universities; and 518 came from municipal universities. 636 of participants were younger than 30, 784 of them were aged between 31 and 40, 355 of them were aged between 41 and 50, 108 of them were older than 50, and 18 of them did not response to age. Approximately 49% of the participants reported 6 to 20 years of teaching experience, approximately 35% reported less than 5 years of teaching experience, and 16% reported more than 20 years of experience as a university teacher.

### Measures

#### Job Stress

Job stress was assessed by the Scale for Occupational Stressors on College Teachers (Li, 2008). The scale contains 64 items with 5-point Likert responses (from “no stress” = 0, to “very serious stress” = 4). The items address the stress with various subscales, namely, organizational factors, interpersonal relations, professional development, workloads, lack of interest, job adaptation, scientific research, teacher–student relationships, and family-related factors. An example item is, “It’s hard to get all kinds of honors.” Cronbach’s alpha reliability coefficient for this scale was 0.975.

#### Job Burnout

Job burnout was measured using the Scale of Job Burnout on University Teachers (Wang and Gao, 2010), which has 37-item with four subscales include organizational depersonalization, reduced personal accomplishment, emotional exhaustion, and researching exhaustion. Response categories ranged from “not at all” = 0, to “always” = 4. Example items include, “When I wake up in the morning, I feel uneasy at the thought of a day’s work.” Cronbach’s alpha reliability coefficient for this scale was 0.959.

#### Job Satisfaction

Job satisfaction was assessed by the revised Chinese version of the Job Satisfaction Scale (Wang et al., 1993), a highly reliable and valid scale that consists of eight items rated on a 5-point scale from 1 for “not at all satisfied” to 5 for “very satisfied.” An example item is, “The current job is commensurate with your expectations of it.” Cronbach’s alpha reliability coefficient for this scale was 0.884.

#### Organizational Commitment

Organizational commitment was measured using the Organizational Commitment Questionnaire (Wu and Yang, 1982). The scale contains 15 items with 5-point Likert responses (from “strongly disagree” = 1 to “strongly agree” = 5). Example items include, “I am proud of being a member of this unit.” Cronbach’s alpha reliability coefficient for this scale was 0.905.

All the above scales have Chinese versions with acceptable reliability and validity when being administered to Chinese university teachers (e.g., Wang et al., 2008, 2011, 2013).

### Statistical Analysis

The hypothesized independent-dependent variables relationship (H1), mediating effects (H2, H3, H4), specific indirect effect (H5) and moderating effect (H6) were all analyzed using structural equation model (SEM) in Mplus 7.0.

The basic function of SEM is to exploring multivariable (including latent variables) relationships. Before testing specific indirect effect and moderating effects, SEM was set up to investigate predicting relations among study variables. Maximum likelihood method (MLM) was utilized to simultaneously examine relationship between variables while bootstrapping method was used to evaluate mediating effects of job burnout and job satisfaction in SEM. Model fit of the SEM was assessed by using an array of fit criteria. Our total sample size was moderately large (*n* = 1906) and chi-square is affected by even trivial deviations with a large sample, which easily suggests a poor fit (Kline, 2010). Therefore, we mainly referred to four additional indicators of goodness of fit, namely, Comparative Fit Index (CFI), Tucker-Lewis index (TLI), Root Mean Square Error of Approximation (RMSEA), and Standardized Root Mean Square Residual (SRMR). Hu and Bentler (1999) recommended that the cutoff values of good model fit are close to 0.95 for indices such as the CFI and TLI, in combination with a value close to or lower than 0.09 for SRMR and values smaller than 0.06 for RMSEA.

## Results

### Data Screening

Before constructing SEM, we need to test the assume of multivariate normality, so skewness and kurtosis were examined to determine the normality of the variables (Song et al., 2014). The distribution of the variables was not far from the normality because the absolute value of skewness was less than 3 and the absolute value of kurtosis was less than 10 (see Table 1) (Kline, 2010; Song et al., 2014).

**TABLE 1:** Normality, means, standard deviations (SDs), and correlations among variables.

	Skewness	Kurtosis	Mean (SD)	Str	Bur	Sat	Com
Str	−0.05	−0.03	109.40 (42.85)	1			
Bur	0.13	−0.51	52.15 (24.17)	0.427**	1		
Sat	−0.57	0.34	28.18 (5.76)	−0.342**	−0.632**	1	
Com	0.25	−0.29	53.46 (9.28)	−0.235**	−0.578**	0.626**	1

Str, job stress; Bur, job burnout; Sat, job satisfaction; Com, organizational commitment. **p < 0.01.

### Descriptive Statistics and Difference Test

The results of means (M), standard deviations (SD) and correlation analysis among four variables were presented in the overall table (see Table 1) and group-specific table (see Supplementary Tables S1, S2). As shown in the table, all the four focal variables were moderately correlated with each other. A one-way ANOVA with a *post hoc* Scheffe test was conducted to examine between-group differences in each type of university (i.e., national, provincial, and municipal). The results indicated statistically significant differences between all the study variables (see Supplementary Table S1).

### Test of Measurement Model and Common Method Bias

Before testing the measurement model, as suggested by statistical scholars (Little et al., 2002; Bandalos, 2008; Wu and Wen, 2011), item parcels were created for each variable to reduce model complexity and estimation errors. We followed the common strategy by reducing the total number of indicators of each construct to three. Exploratory factor analysis (EFA) was first conducted for each of the two variables to test whether both of them were unidimensional, which was the premise of item parceling. Confirmatory factor analysis (CFA) for each scale was then carried out to determine the factor loading values for each item. Next, judging by the factor loading, the items of each scale were divided into three parcels for equalizing the average factor loadings through balance method which belongs to factorial algorithm (Rogers and Schmitt, 2004; Wu and Wen, 2011). Finally, each indicator was created by averaging the items in each parcel. As shown in Table 2, the hypothesized four-factor model displayed the best fit indices and was superior to various alternative models.

**TABLE 2:** Discriminant validity with comparison of alternative measurement models.

Model	χ^2^	df	Δχ^2^	Δdf	RMSEA	SRMR	TLI	CFI
Hypothesized four-factor model (Str, Bur, Sat and Com)	438.79	176	—	—	0.06	0.04	0.98	0.99
Three-factor (Str and Bur combined)	5097.50	189	4658.71***	13	0.24	0.23	0.71	0.73
Two-factor (Str, Bur and Sat combined)	8302.07	199	7863.28***	23	0.30	0.30	0.55	0.55
Single-factor (all variables combined)	9927.35	206	9488.56***	30	0.33	0.20	0.48	0.46

Str, job stress; Bur, job burnout; Sat, job satisfaction; Com, organizational commitment. The χ^2^ difference was compared with the value of the four-factor model. ***p < 0.001.

Since all variables were measure by self-reported scales, common method variance may exist. We detected common method bias by Harman’s single factor test (Harris and Mossholder, 1996). We estimated a CFA model in which the number of latent factors was set to 1. The fitting indexes were as follows: χ^2^/df = 9.166, RMSEA = 0.079, CFI = 0.423, TLI = 0.414, and SRMR = 0.125. The fit of the one latent method model was much worse (Hu and Bentler, 1999), indicating no serious common method bias.

### Test of Hypotheses

**FIGURE 2:**
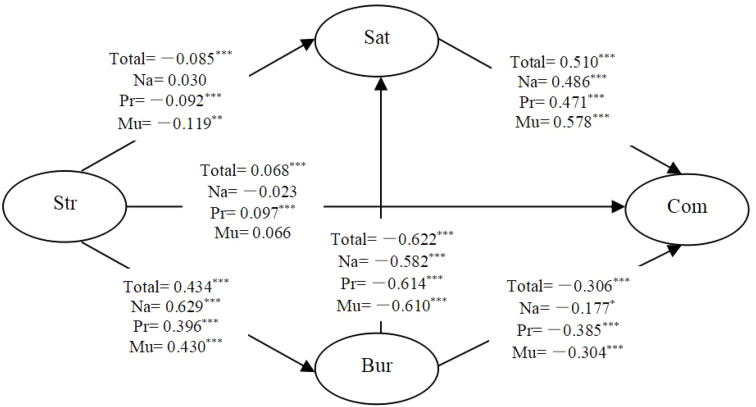
Standardized regression coefficient of entire sample and three groups. Na, national university; Pr, provincial university; Mu, municipal university; Str, job stress; Bur, job burnout; Sat, job satisfaction; Com, organizational commitment. ^∗^*p* < 0.05; ^∗∗^*p* < 0.01; ^∗∗∗^*p* < 0.001.

The SEM analysis indicated that the model achieved a good model fit: CFI = 0.989, TLI = 0.985, SRMR = 0.023, and RMSEA = 0.053. In Figure 2, the standardized path coefficients of entire sample were all significant. Specifically, job stress negatively affected job satisfaction and positively affected job burnout, job satisfaction affected organizational commitment positively and job burnout influenced job satisfaction and organizational commitment negatively.

Hypothesis 1 proposed that job stress could positively predict organizational commitment in line with our expectations, the results presented in Figure 2 indicated job stress influenced organizational commitment positively for the overall sample. Thus, hypothesis 1 was supported.

The mediating effects of job burnout and job satisfaction on the job stress-organizational commitment relationships were investigated by computing from 1,000 bootstrap samples. To avoid Type I errors, bias-corrected (BC) interval was be also chosen (Efron and Tibshirani, 1993). BC bootstrap 95% confidence interval indicated significant mediating effect when zero was excluded. From Table 3 we found the indirect relation of job stress with organizational commitment, as mediated by job burnout and job satisfaction independently and jointly. Thus, hypotheses 2, 3, and 4 were supported.

**TABLE 3:** Mediating effect sizes of job burnout and job satisfaction.

	Estimate	Lower 2.5%	Upper 2.5%
Str→Com	−0.314***	−0.309	−0.234
Str→Bur→Com	−0.133***	−0.143	−0.092
Str→Sat→Com	−0.043***	−0.058	−0.017
Str→Bur→Sat→Com	−0.137***	−0.140	−0.098

Str, job stress; Bur, job burnout; Sat, job satisfaction; Com, organizational commitment. ***p < 0.001.

Specific indirect effect test could figure out mediation effect of each mediator in a multiple mediation model. It is sometimes important to test the hypothesis that two indirect effects—whatever their magnitudes may be—are equal in size (Preacher and Hayes, 2008). We applied bootstrapping method in a SEM context for specific indirect effects test in multiple mediator models. First we defined path a was the effect of X on the proposed mediator (M), while path b was the effect of M on Y partialing out the effect of X. Unstandardized regression coefficients were commonly used to quantify all of these paths. The indirect effect of X on Y through M can be quantified as the product of a and b (i.e., ab). Different mediators were distinguished by subscript numbers (e.g., a1, b1 belonged to M1). Then followed by MacKinnon (2000) and Preacher and Hayes (2008), the contrast indirect effects was computed after Equation fc = a1b1 − a2b2 for each bootstrap resample, and a sampling distribution of this contrast was generated.

Through a specific mediation effect difference test, we found that the independent mediating effect of job burnout was significantly greater than job satisfaction in overall sample (see Table 4). In other words, job burnout may play a key mediate role in the job stress—organizational commitment relationships. Thus, hypothesis 5 was supported.

**TABLE 4:** Unstandardized mediating effect sizes of job burnout and job satisfaction and their differences.

	Estimate	Lower 2.5%	Upper 2.5%
Str→Sat→Com	−0.038***	−0.055	−0.020
Str→Bur→Com	−0.115***	−0.139	−0.095
Test	−0.077***	−0.109	−0.050

Str, job stress; Bur, job burnout; Sat, job satisfaction; Com, organizational commitment. ***p < 0.001.

In provincial universities, the same result of specific mediating effect as the overall sample was observed. However, in national and municipal universities, the specific mediating effect of job satisfaction and job burnout had no significant difference (see Table 5).

**TABLE 5:** Unstandardized mediating effect sizes of job burnout and job satisfaction and the differences between them among the three university types.

	Na			Pr			Mu		
			
	Estimate	Lower 2.5%	Upper 2.5%	Estimate	Lower 2.5%	Upper 2.5%	Estimate	Lower 2.5%	Upper 2.5%
Str→Sat→Com	0.012	−0.037	0.070	−0.035***	−0.059	−0.015	−0.059**	−0.099	−0.024
Str→Bur→Com	−0.074	−0.143	−0.018	−0.117**	−0.143	−0.094	−0.084**	−0.127	−0.042
Test	−0.085	−0.148	0.021	−0.082***	−0.118	−0.048	−0.025	−0.078	0.039

Na, national university; Pr, provincial university; Mu, municipal university; Str, job stress; Bur, job burnout; Sat, job satisfaction; Com, organizational commitment. **p < 0.01; ***p < 0.001.

### Moderating Role of Three Types of University

Before moderating effect test, measurement invariance was first conducted to examine whether the job stress, job burnout, job satisfaction, and organizational commitment were measured in the same way for three types of university teachers. We examined the measurement variance by testing factorial invariance (i.e., testing whether factor loadings are equal across groups). We compared a baseline model where all the factor loadings could vary among the three groups with a measurement model where equality restrictions were placed on the factor loadings. We did not restrict factor variances and co-variances because they were expected to be sample specific, whereas factor loadings should be equal (Rooks et al., 2016). A chi-square test showed that measurement variance was not present.

Then we examined the moderating effects of university types on the relationship among job stress, job burnout, job satisfaction and organizational commitment through multi-group analysis methods. First, in order to determine whether the data of each group fit the structural model well to conduct multi-group analysis, we estimated the baseline model of each group separately. The results showed that all three groups met sufficient fit index (National: RMSEA = 0.036, CFI = 0.995, TLI = 0.993, SRMR = 0.032; Provincial: RMSEA = 0.049, CFI = 0.991, TLI = 0.987, SRMR = 0.022; Municipal: RMSEA = 0.065, CFI = 0.984, TLI = 0.977, SRMR = 0.028). Across the three groups, differences in the structural path coefficients were observed (see Figure 2).

Second, we estimated a model initially in which all the path coefficient was estimated freely across the three groups (M0). In the unconstrained model, all parameters were allowed to be estimated freely across the three groups (M0). For M0, the model matched the data (CFI = 0.986, TLI = 0.984, RMSEA = 0.049, SRMR = 0.032).

Third, we constrained the path estimation to be equal sequentially, one parameter at a time. In the constrained model, each path coefficient was fixed as equal sequentially across the three groups. Chi-square difference test was used to evaluate whether the goodness of fit has significantly different between the constrained models and M0. Additionally, a scaled chi-square test by Satorra (1999) was used when testing the nested models. A significant chi-square test of difference suggests that the less constrained model should be retained, and a non-significant test indicates that the two models provide equal fit to the data. The results indicated that the four paths were different among the three university types: the positive effect of job stress on job burnout between national and provincial universities (Δχ^2^ = 16.297, Δdf = 1, *p* < 0.001), the positive effect of job stress on job burnout between national and municipal universities (Δχ^2^ = 9.166, Δdf = 1, *p* < 0.01), the negative effect of job burnout on organizational commitment between national and provincial universities (Δχ^2^ = 10.876, Δdf = 1, *p* = 0.001), and the negative influence of job burnout on job satisfaction between national and municipal universities (Δχ^2^ = 5.237, Δdf = 1, *p* < 0.05).

## Discussion

Under the framework of the JD-R model, the objectives of this study were to assess the relationships among job stress and organizational commitment as well as the mediating effect of job burnout and job satisfaction, and to examine the moderating role of university types. Overall, the conceptual model was entirely supported by the results from SEM and multi-group analysis. The analysis and interpretations of results were presented as follows.

### Job Stress Could Positively Predict Organizational Commitment

We assumed that job stress could positively predict organizational commitment among our research sample—Chinese university teachers. Within our expectation, the significant positive direct effect between job stress and organizational commitment was observed, that is, an increase in the stress level of university teachers could promote (rather than reduce) their organizational commitment. The result was inconsistent with most previous research findings that the direct effect of job stress to organizational commitment was negative (Jepson and Forrest, 2006) or non-significant (Antón, 2009); but consistent with the study implemented by Kang and Liu (2018) in Chinese sample. This result can be explained in the following three aspects. First and foremost, cultural differences may lead to different perceptions of organizational commitment. Researchers have argued that commitment is a complex attitude influenced by the nature of the groups and contextual contingent (Markus and Kitayama, 1991; Razak et al., 2010). Chinese dominant culture highly values collectivism, and loyalty and commitment to group goals are core features of Chinese collectivist values (Chang and Lu, 2007; Lu et al., 2010). Studies have found that Chinese employees reported a higher-level commitment than their counterparts in other countries (Perrewe et al., 1995; Cheng and Stockdale, 2003; Miembazi and Qian, 2017). In order to take responsibility for the unity and good functioning of the organization, Chinese people are more likely to show a positive side in the face of stress, namely perceiving stress as a challenge rather than burden. In this way, their emotion toward the organization could be enhanced. At the same time, universities have been long described as the Ivory Tower, where people work there enjoy a stable working environment, job security and high social status. Thus, becoming a university teacher has extraordinary significance for most Chinese people, which makes them have more attachment to this profession. For instance, one study showed the organizational commitment of Chinese university teachers is above average (Zhou, 2015). In addition, job stress can be the obstacle as well as the support. Xu and Zheng (2012) put forward that young university teachers’ working efficiency will be enhanced unceasingly even under the stress of difficult task when they obtained proper support from organization. Also, an exploratory study conducted in China identified challenging job demands may have positive effect on university teachers’ well-being (Han et al., 2019).

### The Mediating Effects of Job Burnout and Job Satisfaction

In this study, job burnout was observed to be a mediator in the relationship between job stress and organizational commitment. The effect of job stress to organizational commitment was partially mediated by job burnout, suggesting that university teachers perceiving higher levels of job stress for a long time would feel burnout by their job, and be reluctant to stay in the organization consequently. Researchers have argued that job burnout occurs when an individual’s lost resources are not recovered or incomplete for a long time (Głebocka and Lisowska, 2007), as a result their attitudes toward work will be affected (Cordes and Dougherty, 1993). These opinions were supported in this study.

Job satisfaction was found to act as a mediator in the relationship between job stress and organizational commitment, corresponding to ideas that consider job satisfaction as a prior variable of organizational commitment (e.g., Nagar, 2012; Miembazi and Qian, 2017). This result suggested that university teachers perceiving a higher level of job stress would feel unsatisfied with their job and be reluctant to stay in the organization as before. We also found the serial mediated effect of job burnout and job satisfaction in the mechanism of how job stress influencing organizational commitment. Specifically speaking, when university teachers feel high job stress in the long term, they would feel burnout, and then result in unsatisfying with their job; therefore, their willingness to stay in the organization may be reduced.

Furthermore, a specific mediation effect difference test revealed that the independent mediating effect of job burnout is significantly greater than job satisfaction in the overall sample. As a result, job stress is more likely to affect organizational commitment via job burnout, that is, job burnout may play a key mediate role in the relationship between job stress and organizational commitment.

### The Moderating Effect of University Type

The result of multi-group analysis showed that for national university teachers, the positive effect of job stress on job burnout is the highest among three types of university teachers. National university teachers are burdened by great expectations from the public because they have taken on the mission of university, which is to play exemplary and guiding role in improving pedagogy, scientific research, and social services. To achieve performance appraisal standards and requirements, their resources are always in a state of high consumption. Liu’s (2015) study found that on average, Chinese university teachers work 52 h per week, which is much higher than the legal weekly working hours (44 h); even during vacations, they work 32.9 h per week. Additionally, the average working hours of 211 project university (a category of national university) teachers are longer than their non- 211 project counterparts. This phenomenon perhaps explains why national university teachers experience a higher degree of job stress and are more prone to burnout. Compared with counterparts, national university teachers have a high level of job stress; however, they could enjoy higher salaries, more advantageous research conditions, easier access to necessary resources and other benefits. Maybe these resources that they enjoy could alleviate their negative attitude toward work and organization. As the results of this study displayed, the negative effect of job burnout on organizational commitment for teachers in national university is lower compared with those who in provincial university; and the negative effect of job burnout on job satisfaction for teachers in national university is lower compared with those who in provincial university.

We also observed that only the job stress of provincial university teachers can significantly positively predict their organizational commitment, that is, the positive direct effect from job stress to organizational commitment may be mainly contributed by provincial university teachers. Provincial university teachers enjoy medium-quality scientific research conditions and welfare treatment, they have vastly potential for development, so they work hard to catch up with national university teachers. However, the actual situation is that they received the least educational output. For example, the Annual Report of China’s Education indicated that the employment rate of provincial university students (67.4%) is much lower than that of national university students (75.5%) and municipal university students (78.1%) (Yang, 2014). According to Festinger’s (1957) cognitive dissonance theory, when an individual’s attitudes are inconsistent with their actions, cognitive dissonance occurs. Since cognitive dissonance can produce mental tension, individuals will strive to eliminate this tension to restore cognitive balance. As for provincial university teachers, there is a conflict between attitude and action perhaps they can only change their attitude toward work place to restore cognitive balance, namely put love organization as a reason for high stress work, in this way organizational commitment increased with stress. At the same time, through the specific mediation effect difference test, we found that only in provincial universities the independent mediating effect of job burnout is significantly greater than job satisfaction. That is to say, their organizational support mismatched development ambition, and their input is disproportionate with output, which are more likely to make them job burnout, rather than job satisfaction.

### Implications

This paper innovatively compared different types of samples in the relationship between job stress and organizational commitment under non-western culture context. Although the theories and methods in this paper were from western, the questionnaires were developed by Chinese scholars and participants were selected from China, which were more suitable for China’s indigenization research. This study also extended the JD-R model with the sample of Chinese university teachers, and introduced a new kind of job resources—university types, which accumulates empirical evidence for cross-cultural research on organizational behavior field in the future.

The current research has its implication in organizational psychology. University administrators can implement measures to stimulate teachers’ positive work attitude to reduce job burnout, and provide effective interventions to enhance job satisfaction and organizational commitment. Interventions can be taken to avoid the feeling of burnout, such as showing the effects of stress and techniques to cope with occupational stress, which is proved effective (Wu et al., 2006). Overall, the result of this study showed that the higher the job stress perceived, the stronger the organizational commitment would be. Therefore, it is good for university teachers to maintain a moderate work pressure. Besides, university type plays an important role among the relationship of the variables, education organization could take measures from the view of improving resource allocation. To be more specific, as for provincial universities, striking a balance between resources and tasks is an effective way to reduce job burnout, which can further increase employee loyalty to the university. As for municipal universities, optimizing resource allocation is a good way to enhance teachers’ job satisfaction. Fortunately, the problem of uneven distribution of higher education resources has been put on the agenda in National People’s Congress (NPC, the highest organ of state power of the People’s Republic of China), and this gap will be narrowed for the foreseeable future.

### Limitations and Future Research

There are two limitations in the present study. First, this study only examined the moderating role of three types of university, and pointed out that resource difference may be the reasons for groups’ different presented in the study model. We suggest future studies to include other potential organizational factors, such as organizational climate and leadership style to explain these differences in a more comprehensive way. Second, we have studied the influence of job stress on job satisfaction, job burnout and organizational commitment under the guidance of previous research, but it is also possible that one’s organizational commitment affects feelings of job stress. Hence, cross-lagged longitudinal design should be further considered to infer causality among variables.

## Data Availability Statement

The raw data supporting the conclusions of this article will be made available by the authors, without undue reservation.

## Ethics Statement

The studies involving human participants were reviewed and approved by the Human Research Ethics Committee of Shandong Normal University. The patients/participants provided their written informed consent to participate in this study.

## Author Contributions

PW conceived this study. PC designed and completed the manuscript. JW participated in data analysis. RP polished the language. YS, MY, and LJ participated in data collection. XZ submitted and revised the manuscript. DZ provided significant advice in revision process. All authors contributed to the article and approved the submitted version.

## Conflict of Interest

The authors declare that the research was conducted in the absence of any commercial or financial relationships that could be construed as a potential conflict of interest.
