# Supplementary material for: Association Between Job Stress and Organizational Commitment in Three Types of Chinese University Teachers: Mediating Effects of Job Burnout and Job Satisfaction
Source: Front Psychol. 2020 Oct 8;11:576768. doi: 10.3389/fpsyg.2020.576768 (PMC7578428; doi:10.3389/fpsyg.2020.576768)
Supplement: Supplementary file 1 [file Table_1.DOCX]

Supplementary Material

# Supplementary Tables

**Table 1 |** Differences in the variables among the three types of university

|  | Na  Mean (SD) | Pr  Mean (SD) | Mu  Mean (SD) | F | post hoc |
| --- | --- | --- | --- | --- | --- |
| Str | 115.51 (40.73) | 105.27 (42.48) | 115.83 (43.56) | 13.75*** | M_Na_, M_Mu_>M_Pr_ |
| Bur | 61.88 (22.99) | 48.57 (23.40) | 55.72 (24.66) | 38.62*** | M_Na_ >M_Mu_>M_Pr_ |
| Sat | 26.47 (5.01) | 29.15 (5.35) | 26.78 (6.44) | 43.87*** | M_Pr_>M_Na_, M_Mu_ |
| Com | 50.46 (7.77) | 54.35 (9.42) | 52.83 (9.27) | 19.09*** | M_pr_>M_Mu_>M_Na_ |

*Note.* n = 1524. SD: standard deviation. Na: national university, Pr: provincial university, Mu: municipal university; Str: job stress, Bur: job burnout, Sat: job satisfaction, Com: organizational commitment. ****p* < 0.001.

**Table 2 |** Correlations among job stress, job burnout, job satisfaction, and organizational commitment among the three university types

|  | Na | | | |  | Pr | | | |  | Mu | | | |
| --- | --- | --- | --- | --- | --- | --- | --- | --- | --- | --- | --- | --- | --- | --- |
|  | Str | Bur | Sat | Com |  | Str | Bur | Sat | Com |  | Str | Bur | Sat | Com |
| Str | 1 |  |  |  |  | 1 |  |  |  |  | 1 |  |  |  |
| Bur | 0.615** | 1 |  |  |  | 0.385** | 1 |  |  |  | 0.402** | 1 |  |  |
| Sat | -0.312** | -0.503** | 1 |  |  | -0.318** | -0.622** | 1 |  |  | -0.349** | -0.648** | 1 |  |
| Com | -0.236** | -0.441** | 0.593** | 1 |  | -0.230** | -0.602** | 0.626** | 1 |  | -0.209** | -0.540** | 0.627** | 1 |

*Note.* Na: national university, Pr: provincial university, Mu: municipal university; Str: job stress, Bur: job burnout, Sat: job satisfaction, Com: organizational commitment. ***p* < 0.01.
